# Supplementary material for: Incidence and treatment trends of infectious spondylodiscitis in South Korea: A nationwide population-based study
Source: PLoS One. 2023 Jun 29;18(6):e0287846. doi: 10.1371/journal.pone.0287846 (PMC10309630; doi:10.1371/journal.pone.0287846)
Supplement: Attachment — Submitted filename: reviewers comment.docx [file pone.0287846.s001.docx]

It is a useful data from big sample.

P4 line 68; Please explain about the abbreviation “TS”

P17 line 222；Provide citations for“several study”

Discussion

In the present results, women were more numerous and the age group with the highest number of respondents in their 50s. Can you please add further discussion on these matters?
